# Bullying victimization, suicidal behavior, and help-seeking in Chinese adolescents: a legal-psychological Study of resilience and responsibility

**DOI:** 10.3389/fpsyg.2025.1625188

**Published:** 2025-12-08

**Authors:** Xianyun Ge

**Affiliations:** School of Law, Yangzhou University, Yangzhou, Jiangsu, China

**Keywords:** bullying victimization, suicidal behavior, help-seeking attitudes, resilient coping, peer support, legal responsibility, moral autonomy

## Abstract

Bullying victimization is a significant public health concern among adolescents, with profound psychological and behavioral consequences. Grounded in an ecological and ethical framework, this study connects psychological mechanisms with the moral and institutional responsibilities involved in protecting adolescents’ wellbeing. It examines the associations between bullying victimization, suicidal behavior, help-seeking attitudes, and resilient coping among a sample of 987 middle school students (aged 12–15) in China. Additionally, it explores the mediating roles of peer support and core self-evaluations in these associations. Data were analyzed using SmartPLS with the PROCESS module. The results indicate that bullying victimization is positively associated with suicidal behavior, and negatively associated with help-seeking attitudes and resilient coping. Core self-evaluations significantly mediate the associations between bullying victimization and both help-seeking attitudes and resilient coping, highlighting the importance of self-perceptions in mitigating adverse effects. Peer support, while positively related to help-seeking attitudes and resilient coping, did not significantly mediate the association between bullying victimization and suicidal behavior. These findings suggest that individual psychological resources play a crucial role in shaping adolescents’ responses to bullying, while social support alone may not be sufficient to buffer the risk of suicidality. The study underscores the need for multi-level and culturally sensitive interventions, integrating school-based mental health programs, counseling services, and family support initiatives to strengthen resilience and promote help-seeking among adolescents.

## Introduction

Bullying victimization is a pervasive issue affecting adolescents worldwide, with significant psychological, social, and academic consequences. Among Chinese adolescents, the prevalence of bullying remains a pressing concern, with various studies highlighting its impact on mental health and overall wellbeing. Victimized adolescents often experience heightened levels of psychological distress, including depression, anxiety, and suicidal ideation, while also struggling with social integration and academic performance. These effects raise critical questions regarding how bullying is associated with key psychological constructs such as suicidal behavior, help-seeking attitudes, and resilient coping (Chen and Xiao, 2025; Santoso, 2024; Zhou et al., 2024).

Research suggests that adolescents’ ability to cope with bullying varies significantly and is influenced by personal characteristics and social support structures. Core self-evaluations, which encompass self-esteem, emotional stability, and self-efficacy, are crucial in shaping how individuals respond to victimization. Similarly, peer support has been identified as a potential protective factor that can mitigate the adverse consequences of bullying. However, the extent to which these factors mediate the associations between bullying victimization and mental health outcomes remains unclear (Liu et al., 2024; Peng et al., 2024).

This study focuses on a sample of Chinese adolescents aged 12–15, a developmental stage characterized by heightened vulnerability to peer victimization and its psychological effects. By examining the associations between bullying victimization, suicidal behavior, help-seeking attitudes, and resilient coping, this research aims to provide a comprehensive understanding of how adolescents navigate these challenges. Additionally, the study explores the mediating roles of peer support and core self-evaluations in shaping these associations, offering insights into potential intervention strategies (Li et al., 2023; Zhang Y. et al., 2024).

Grounded in an ecological and moral-legal framework, this study further considers how adolescent wellbeing is influenced not only by individual and social factors but also by institutional and ethical responsibilities within school contexts. This interdisciplinary lens integrates psychological, educational, and legal perspectives to better capture the complexity of bullying and its consequences. The findings of this study have important implications for educators, mental health professionals, parents, and policymakers. Understanding the psychological mechanisms underlying bullying victimization can inform the development of targeted interventions aimed at reducing the negative effects of victimization and fostering adaptive coping strategies. Addressing individual and social factors, this research seeks to contribute to the broader goal of promoting adolescent wellbeing and creating safer school environments. Ultimately, the study underscores the importance of integrating evidence-based and ethically informed approaches to support victimized adolescents and prevent the long-term consequences of bullying (Ferraz De Camargo et al., 2023; Halliday et al., 2021; Hutson and Mazurek Melnyk, 2022; Schoeler et al., 2018).

## Literature review

### The relationship between bullying victimization and suicidal behavior among Chinese adolescents

From a developmental-ecological and moral-legal perspective, bullying victimization represents a major contextual and ethical challenge that shapes adolescents’ psychological wellbeing. Rather than implying causality, prior research has shown that bullying victimization is associated with a higher risk of suicidal behavior, particularly among adolescents. Studies on Chinese adolescents aged 12 –15 indicate that experiencing bullying—whether physical, verbal, or cyber—is related to an increase the likelihood of suicidal ideation and attempts. A recent study conducted in Yixing City, China, examined the association between different forms of bullying and suicidal ideation, highlighting that poly-victimization (experiencing multiple types of bullying) is especially linked to suicidal thoughts (Sheng et al., 2024).

Psychological distress plays a mediating role in this association. Adolescents who experience bullying report higher levels of depression, anxiety, and social withdrawal, all of which contribute to increased suicidal risk (Zhao and Yao, 2022). The relationship between bullying and suicidality is particularly concerning, given that victims often suffer in silence, lacking access to adequate mental health resources. A study conducted in Zhejiang Province found that both traditional and cyberbullying victimization and cyberbullying victimization were associated with elevated suicidal ideation and attempts, with cyberbullying exerting a stronger association due to its pervasive and relentless nature (Wang H. et al., 2023).

In sum, empirical research strongly supports the hypothesis that bullying victimization is positively associated with suicidal behavior among Chinese adolescents. The interplay of psychological distress, sleep disturbances, and gender differences highlights the complexity of this issue and the urgent need for targeted interventions and ethical responsibility within educational settings to address the psychological harm associated with bullying.

### The relationship between bullying victimization and help-seeking attitudes among Chinese adolescents

From an ecological and moral-legal standpoint, adolescents’ willingness to seek help when facing bullying reflects not only individual coping capacities but also the ethical and institutional contexts that shape their autonomy and access to support. Help-seeking is a crucial coping mechanism for adolescents experiencing bullying victimization. The extent to which bullied adolescents seek help depends on various psychological, social, and cultural factors. Recent research on Chinese adolescents suggests that victimization experiences are associated with their willingness to seek support from peers, teachers, family members, or mental health professionals (Xie et al., 2022).

Studies indicate that bullied adolescents in China often adopt different help-seeking strategies based on the severity and type of victimization they experience. In particular, emotional distress and depression appear to mediate the associations between bullying and help-seeking behaviors. A study on Chinese school students found that while some victims actively seek emotional support, others may withdraw due to feelings of shame or fear of retaliation (Huang et al., 2023).

Additionally, social support plays a vital role in help-seeking behaviors. Adolescents who perceive strong peer and familial support tend to have more positive attitudes toward seeking help. A study found that students who experienced bullying but had a strong support network were more likely to report their victimization and seek assistance from trusted adults (Chen et al., 2022). Recent reviews of the literature on bullying and help-seeking emphasize that adolescents’ responses are shaped by both cultural and structural barriers. For instance, studies in East Asian contexts have shown that norms of emotional restraint, fear of losing face, and hierarchical school relationships often discourage direct requests for help, even when distress is severe (Baek et al., 2023). These findings suggest that help-seeking is embedded in a broader social ecology where legal protection policies and ethical school practices can facilitate or inhibit disclosure.

Recent international studies have addressed help-seeking in the context of bullying victimization. For instance, Zubrick et al. (2020) found that nearly 45% of Australian adolescents who experienced bullying did not seek help, and that social isolation and poor prosocial skills were key determinants of non-help-seeking. Similarly, Grüne and Willems (2024) reported that in a German sample, family cohesion and self-efficacy were positively associated with help-seeking behaviors, while formal help-seeking remained markedly low. In Canada, Kim and Craig (2024) highlighted that anticipated outcomes such as shame, retaliation, and stigma significantly influenced whether bullied adolescents sought help. Collectively, these findings suggest that help-seeking is shaped by a complex interplay of individual perceptions, social support, and institutional contexts, emphasizing the need to frame bullying victimization within a broader ecological and moral-legal perspective.

Despite the positive association between bullying victimization and help-seeking attitudes, barriers still exist. Cultural stigmas surrounding mental health, confidentiality concerns, and fear of social consequences often prevent adolescents from seeking the necessary support. Interventions aimed at reducing these barriers could play a crucial role in improving the psychological wellbeing of bullied adolescents (Kwan et al., 2022). Overall, the research supports the hypothesis that bullying victimization is positively associated with help-seeking attitudes among Chinese adolescents.

### The relationship between bullying victimization and resilient coping among Chinese adolescents

Resilient coping can be understood within a broader ecological and ethical framework that views adolescents as active agents capable of moral growth and adaptation when facing adversity. It refers to an individual’s ability to adapt and recover from adversity, including bullying victimization, effectively. Research on Chinese adolescents suggests that while bullying can have detrimental psychological effects, some victims develop resilience as a coping mechanism. A study investigating the association between bullying victimization and adolescent wellbeing in China found that resilience acted as a protective factor, allowing victims to maintain psychological stability despite negative experiences (Zhang Y. et al., 2024).

Resilience appears to moderate the associations between bullying victimization and emotional distress. Adolescents with higher resilience levels report lower levels of anxiety and depression, even when experiencing bullying (Fang et al., 2022). The ability to reframe negative experiences, seek social support, and develop effective coping strategies enables some bullied adolescents to navigate adversity more effectively.

Additionally, studies indicate that school environments play a significant role in fostering resilience among victims of bullying. Research on resilience in Chinese adolescents suggests that schools promoting supportive peer interactions and emotional regulation strategies contribute to higher resilience among bullied students (Lin et al., 2022). Furthermore, social support has been found to strengthen resilient coping among adolescents facing bullying victimization. A study on school bullying among Chinese adolescents emphasized the role of positive peer relationships in enhancing resilience and reducing the negative psychological impact of bullying (Chen et al., 2022).

Beyond its psychological meaning, resilience also reflects the adolescent’s emerging sense of moral autonomy and responsibility—the ability to respond constructively to harm while preserving ethical agency within the social environment. To sum up, empirical evidence supports the hypothesis that bullying victimization is positively associated with resilient coping among Chinese adolescents. While victimization presents challenges, resilience enables some adolescents to mitigate the negative effects of bullying and develop adaptive coping strategies.

### The mediating role of peer support and core self-evaluations

Recent research framed within ecological and moral-developmental approaches has emphasized that both social context and personal agency influence how adolescents interpret and respond to victimization. Emerging studies highlight the importance of peer support and core self-evaluations in mediating the associations between bullying victimization and its psychological outcomes. Core self-evaluations, which include self-esteem, self-efficacy, and emotional stability, influence how adolescents perceive and respond to bullying experiences. Beyond their psychological function, these self-evaluations are also linked to adolescents’ sense of autonomy and ethical responsibility, reflecting their capacity to act as moral agents within school and peer environments. Studies suggest that positive core self-evaluations can buffer against the negative psychological impact of victimization, reducing suicidal ideation and fostering greater openness to seek help (Gu et al., 2024).

Peer support also plays a crucial role in shaping adolescent responses to bullying. Research has demonstrated that adolescents with strong peer networks exhibit higher resilience and are more likely to use adaptive coping strategies (Ma et al., 2024). Additionally, supportive peer environments encourage victims to seek help and share their experiences, mitigating the mitigating the associations between bullying and poorer mental health outcomes (He D. et al., 2024). Furthermore, studies indicate that core self-evaluations mediate the links between bullying victimization and emotional wellbeing. Adolescents with higher core self-evaluations report lower levels of distress and greater resilience following bullying incidents (Wang L. et al., 2023). These findings underscore the necessity of fostering both peer support and positive self-evaluations in anti-bullying interventions to enhance adolescent coping, resilience, and moral growth.

Hence, based on the above-revised literature, the present study is aimed to test the following hypotheses:

Hence, based on the above-reviewed literature, the present study aims to test the following hypotheses:

*H1*: Bullying victimization is positively associated with suicidal behavior.

*H2*: Bullying victimization is positively associated with help-seeking attitudes.

*H3*: Bullying victimization is positively associated with resilient coping.

*H4*: Peer support and core self-evaluations mediate the associations between bullying victimization and suicidal behavior, help-seeking attitudes, and resilient coping.

**FIGURE 1:**
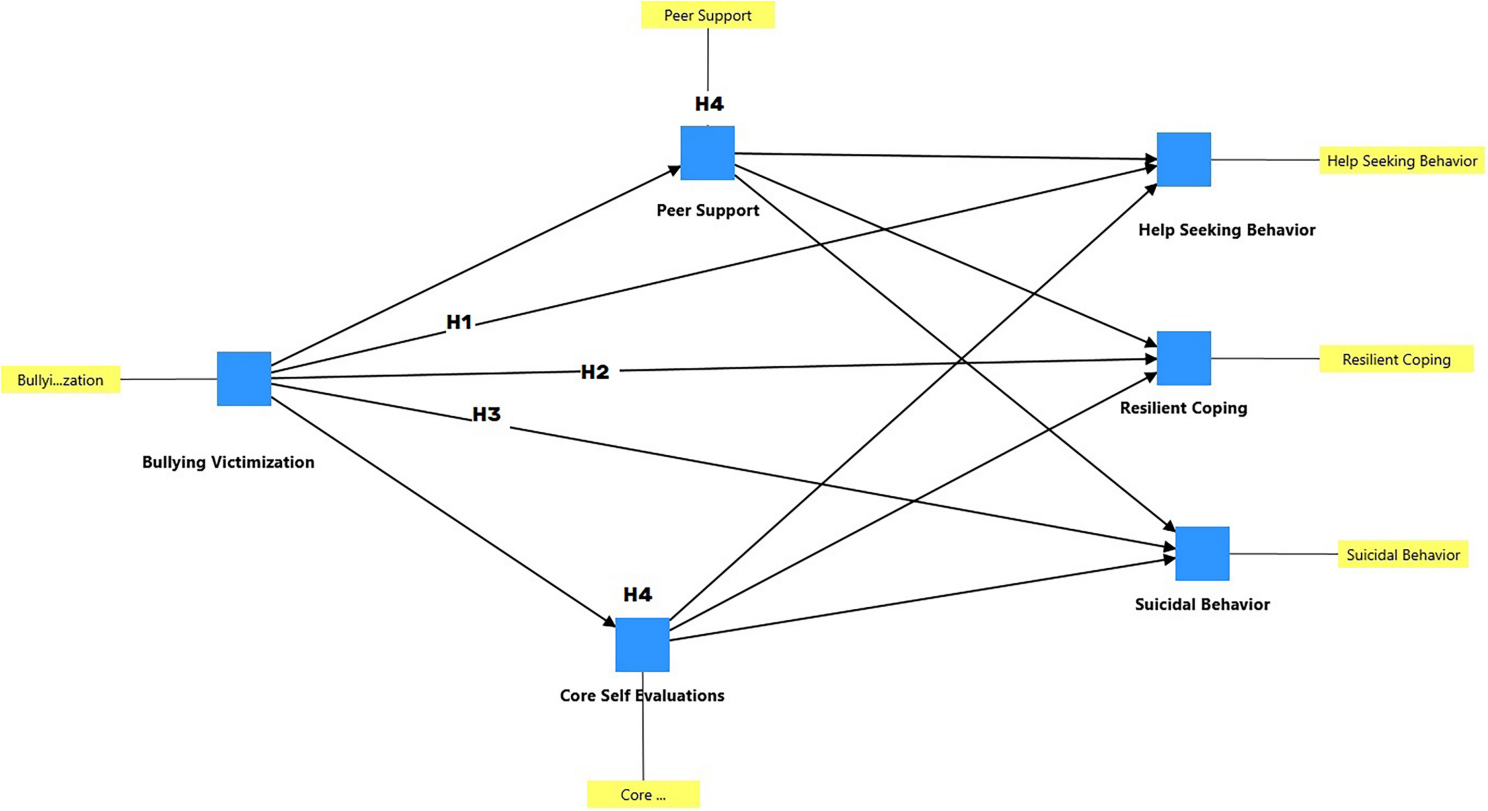
The theoretical model with hypotheses.

The theoretical model for this study is displayed in Figure 1.

## Materials and methods

### Participants

The study included 987 middle school students aged 12–15 from China. The sample’s gender distribution comprised 585 male students (59.3%) and 402 female students (40.7%). No participants selected a “prefer not to say” option, reflecting the binary gender categorization commonly used in Chinese educational contexts. Participants were evenly distributed across the four age groups, with 24.9% aged 12, 26.0% aged 13, 25.4% aged 14, and 23.6% aged 15. The sample also reflected a balanced distribution across grade levels, with 31.1% in seventh grade, 32.2% in eighth grade, and 36.7% in ninth grade.

Family structure varied among participants. The majority, 76.1%, lived with both parents, while 13.9% lived in single-parent households. An additional 8.0% resided with guardians, such as grandparents or extended family members. A smaller proportion, 2.0%, were in alternative living arrangements, including state or private foster care and boarding schools.

Regarding self-rated academic performance, 24.7% of participants classified their performance as below average, while 67.6% reported average performance. Only 7.7% rated their academic achievement as above average.

### Procedure

This study employed a convenience sampling strategy, recruiting participants from middle schools in China. Before data collection, a power analysis was conducted to determine the appropriate sample size for statistical validity. The analysis was based on previous research examining the relationship between bullying victimization and suicidal behavior among adolescents, ensuring sufficient statistical power (≥ 0.80) to detect meaningful effects. Based on these calculations, the target sample size was set at approximately 900 participants, with the final sample comprising 987 students, exceeding the minimum required for robust inferential analysis.

Given that the participants were minors, rigorous ethical safeguards were implemented. The study received approval from the Research Ethics Committee at Yangzhou University, with the approval number LAW.XG23. 00505 and was conducted following the Declaration of Helsinki. In compliance with Chinese legal and ethical standards for research involving minors, informed consent was obtained from parents or guardians and the participating students prior to enrollment. Parents were informed about the study through school communication channels and were provided digital consent forms, which they signed electronically. Additionally, students provided written assent before participation, confirming their willingness to participate.

Before administering the survey, class head teachers received standardized training conducted by school moral education instructors. This training covered the ethical handling of sensitive topics, confidentiality measures, and ensuring a non-coercive and psychologically safe environment during data collection. The teachers then facilitated the survey in a classroom setting, ensuring that students completed it individually and anonymously via a secure web-based platform.

The survey was hosted on a protected online system, accessible through school-provided computers or personal mobile devices. Students were not required to provide identifying information to ensure privacy and data protection, and individual responses remained untraceable. The system was configured to prevent multiple submissions from the same respondent. Participation was voluntary, and students were explicitly informed that they could withdraw from the survey without academic or disciplinary consequences.

Given the sensitive nature of the study, specific mental health safeguards were implemented. Before completing the survey, students were provided with detailed information about available psychological support services and school counseling resources. At the end of the survey, a debriefing message was displayed, reiterating the availability of professional support for students experiencing emotional distress. Additionally, if any student showed distress during the survey, class head teachers were instructed to refer them to the school counseling office for further assistance.

### Instruments

*Bullying victimization* was assessed using a modified version of the Olweus Bully/Victim Questionnaire (Olweus, 1996), which included ten key items covering the frequency, types, and location of bullying incidents. Respondents rated their experiences on a Likert-type scale ranging from 1 (*Never*) to 5 (*Several times a week*). The questionnaire captured various forms of victimization, including verbal, relational, physical, and cyberbullying. Examples of items are: How often have you been bullied at school in the past couple of months?; I was bullied with mean names or comments about my race or color.

*Attitudes toward seeking professional psychological help* were measured using the Brief Version of the Attitudes Toward Seeking Professional Psychological Help Scale (Elhai et al., 2008). This ten-item scale assessed respondents’ willingness to seek psychological counseling in distress. Items were rated on a Likert-type scale ranging from 1 (*Strongly disagree*) to 5 (*Strongly agree*), with higher scores reflecting more positive attitudes toward seeking professional mental health support. Examples of items are “If I believed I was having a mental breakdown; my first inclination would be to get professional attention”; “Considering the time and expense involved in psychotherapy, it would have doubtful value for a person like me” (reversed score).

*Resilient coping* was measured using the Brief Resilient Coping Scale developed by Sinclair and Wallston (2004). This four-item scale evaluates individuals’ perception of their ability to cope with difficulties. Respondents rated their agreement with statements such as “I look for creative ways to cope with difficult situations” on a Likert-type scale ranging from 1 (*Strongly disagree*) to 5 (*Strongly agree*). The Chinese scale version has demonstrated strong reliability and validity (Fung, 2020). The aggregated ratings were used to compute the resilient coping indicator.

*Suicidal behavior* was assessed using the Suicidal Behaviors Questionnaire-Revised (Huen et al., 2022). This four-item instrument measures past suicidal ideation, frequency of suicidal thoughts, disclosure of suicidal intent, and the likelihood of future suicidal behavior. Each item was rated on a Likert-type scale ranging from 1 (*Never*) to 5 (*Very likely*), with higher scores indicating greater suicide risk. Examples of items are: “Have you ever thought about or attempted to kill yourself?”; “How likely is it that you will attempt suicide someday?”.

*Perceived peer support* was evaluated using the Multidimensional Scale of Perceived Social Support (Chou, 2000), specifically the peer support subscale. This subscale included four items assessing participants’ perceptions of their friends’ supportiveness and availability. Responses were measured on a Likert-type scale ranging from 1 (*Strongly disagree*) to 5 (*Strongly agree*), with higher scores indicating stronger perceived peer support. Examples of items are: “My friends really try to help me”; “I have friends with whom I can share my joys and sorrows.”

*Core self-evaluations* were measured using a validated 12-item scale adapted from Judge et al. (2003). This scale assessed self-esteem, self-efficacy, emotional stability, and locus of control. Statements such as “I am confident I get the success I deserve in life” were rated on a Likert-type scale ranging from 1 (*Strongly disagree*) to 5 (*Strongly agree*). The one-factor structure of this measure has demonstrated strong convergent and discriminant validity in multiple studies, including its validation in China (Sun and Jiang, 2017).

Participants provided demographic information, including age, gender (male, female, or prefer not to say), grade level, family structure (living with both parents, living with a single parent, living with guardians, or other), and self-rated academic performance (below average, average, or above average). These demographic variables were included to contextualize the study findings and explore potential response variations based on individual characteristics.

### Data analytic strategy

The data analysis was conducted using SmartPLS 4.1.1.1, which enables Partial Least Squares Structural Equation Modeling (PLS-SEM) and includes the PROCESS module for mediation analysis. This approach was selected because it allows testing complex mediation models and provides consistent estimates even when data deviate from multivariate normality. Prior to analysis, multicollinearity and normality checks were performed; all variance inflation factor (VIF) values were below 5, and skewness and kurtosis values were within acceptable ranges. First, descriptive statistics and frequency distributions were computed to summarize the sample’s demographic characteristics, including gender, age, family structure, and academic performance. Second, correlational analyses were conducted to examine the bivariate relationships between bullying victimization, suicidal behavior, help-seeking attitudes, resilient coping, core self-evaluations, and peer support.

PLS-SEM was applied using the PROCESS module in SmartPLS to test the hypothesized associations. This method was justified by its robustness for models including multiple mediators and smaller sample sizes, its ability to minimize small-sample bias, and its greater statistical power for estimating indirect effects. The bootstrapping procedure (5,000 resamples) was performed to estimate confidence intervals and test the significance of indirect effects. The study focused on Model 4 of PROCESS, which allows for examining simple mediation effects. Model evaluation followed standard PLS-SEM guidelines, including the assessment of model fit indices such as the standardized root mean square residual (SRMR) and the normed fit index (NFI), which were later reported in the Results section. Missing data were handled using listwise deletion, and all items were coded on a five-point Likert scale (1 = strongly disagree to 5 = strongly agree). The analysis also included R^2^ and f^2^ calculations to assess explained variance and effect size.

## Results

### Descriptive statistics and correlations

The means and standard deviations for the key variables indicate moderate levels of bullying victimization, core self-evaluations, resilient coping, suicidal behavior, and help-seeking attitudes. Pearson correlation analysis revealed significant associations among the study variables. As Table 1 shows, bullying victimization was negatively correlated with resilient coping, core self-evaluations, peer support, and help-seeking attitudes. Additionally, suicidal behavior showed negative correlations with resilient coping, core self-evaluations, peer support, and help-seeking attitudes, suggesting that psychological and social support factors may mitigate the risk of suicidal behavior. Notably, bullying victimization had a small but positive correlation with suicidal behavior, indicating that victimized adolescents may be more likely to engage in self-harm.

**TABLE 1:** Descriptive statistics and correlation matrix.

Variable	M	SD	1	2	3	4	5	6
1. Bullying victimization	1.96	0.67	*0.76*	*0.78*	*0.82*	*0.89*	*0.74*
2. Core self-evaluations	3.80	0.62	−0.157				
3. Peer support	3.94	0.67	−0.114	0.548			
4. Help-seeking attitudes	3.83	0.49	−0.197	0.319	0.287		
5. Suicidal behavior	2.31	0.53	0.136	−0.172	−0.089	−0.243	
6. Resilient coping	3.94	0.67	−0.216	0.487	0.428	0.544	−0.283	*0.77*

N = 987. Correlations are significant at *p* < 0.05. Values in the diagonal are Cronbach’s alphas.

### Hypotheses testing

As Table 2 shows, the findings support the hypothesis that bullying victimization is positively associated with suicidal behavior. Adolescents who experience higher levels of victimization report an increased likelihood of suicidal tendencies. The total effect analysis confirms that this relationship remains significant, even after considering potential mediating effects.

**TABLE 2:** Path coefficients.

Path	Coefficients	SD	T-statistics
Bullying victimization → peer support	−0.112	0.037	3.110**
Bullying victimization → core self-evaluations	−0.145	0.037	3.949***
Bullying victimization → resilient coping	−0.100	0.021	4.652***
Bullying victimization → help-seeking attitudes	−0.106	0.025	4.281***
Bullying victimization → suicidal behavior	0.089	0.026	3.407***
Core self-evaluations → resilient coping	0.270	0.025	10.989***
Core self-evaluations → help-seeking attitudes	0.166	0.031	5.415***
Core self-evaluations → suicidal behavior	−0.136	0.036	3.847***
Peer support → resilient coping	0.165	0.023	7.007***
Peer support → help-seeking attitudes	0.112	0.028	4.082***
Peer support → suicidal behavior	0.008	0.034	0.270*ns*

*N* = 987. Significance levels:

***p < 0.001,

**p < 0.01, ns, not significant.

Contrary to expectations, bullying victimization was negatively associated with help-seeking attitudes. This suggests that adolescents who experience victimization may be less inclined to seek support, possibly due to stigma, distrust, or feelings of helplessness. The negative association indicates that victimized individuals may perceive formal or informal support systems as ineffective or inaccessible.

The results indicate that bullying victimization is negatively associated with resilient coping. Rather than fostering resilience, victimization weakens an individual’s ability to adopt adaptive coping mechanisms. This finding suggests that continuous exposure to victimization might erode psychological resilience, reducing the capacity to engage in effective coping strategies when faced with adversity.

**FIGURE 2:**
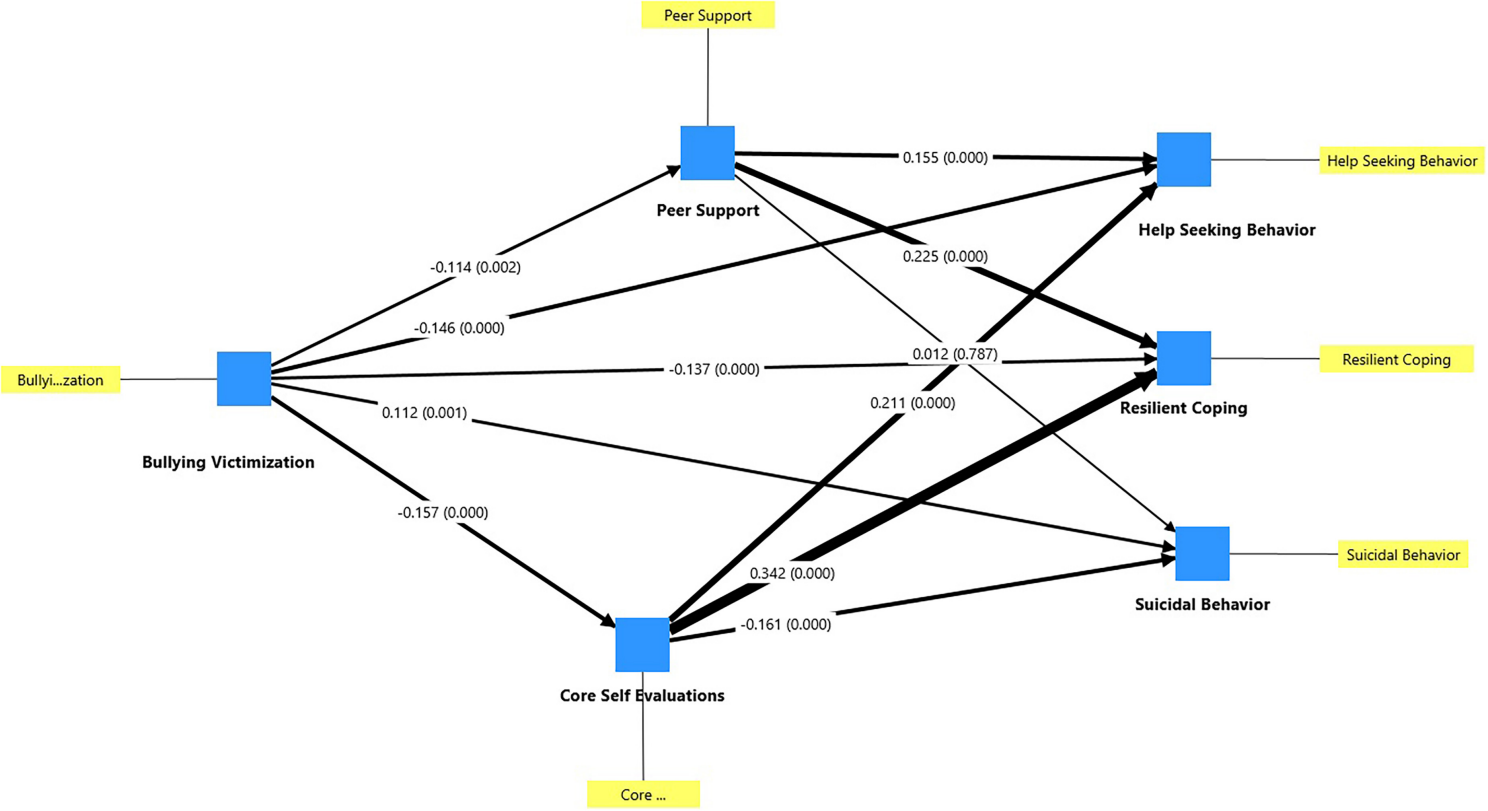
Standardized path coefficients and *p*-values.

As Table 3 shows, the mediation analysis highlights the crucial role of peer support and core self-evaluations in shaping the impact of bullying victimization. Core self-evaluations significantly influence help-seeking attitudes and resilient coping, demonstrating that higher self-worth and emotional stability can buffer the adverse effects of victimization. Similarly, peer support was positively associated with help-seeking attitudes and resilient coping, reinforcing the importance of social networks in mitigating the psychological impact of victimization. However, peer support did not significantly mediate the relationship between bullying victimization and suicidal behavior, indicating that other mechanisms may be involved in this association. Figure 2 displays the standardized path coefficients as well as the *p*-values.

**TABLE 3:** Total and indirect effects.

Path	Sample mean (M)	SD	T-statistics
Bullying victimization → resilient coping	−0.158	0.028	5.600***
Bullying victimization → core self-evaluations	−0.145	0.037	3.949***
Bullying victimization → help-seeking attitudes	−0.143	0.028	5.024***
Bullying victimization → peer support	−0.112	0.037	3.110**
Bullying Victimization → Suicidal Behavior	0.107	0.026	4.055***
Core self-evaluations → resilient coping	0.270	0.025	10.989***
Core self-evaluations → help-seeking attitudes	0.166	0.031	5.415***
Core self-evaluations → suicidal behavior	−0.136	0.036	3.847***
Peer support → resilient coping	0.165	0.023	7.007***
Peer support → help-seeking attitudes	0.112	0.028	4.082***
Peer support → suicidal behavior	0.008	0.034	0.270*ns*

*N* = 987. Significance levels:

***p < 0.001,

**p < 0.01, ns, not significant.

As Table 4 shows, The R^2^ values assess the proportion of variance the model explains in predicting each dependent variable. The results indicate that Resilient Coping has the highest explained variance, suggesting that the predictors significantly contribute to this outcome. Help-seeking attitudes also exhibit a substantial explained variance, while Suicidal Behavior shows a lower R^2^ value, implying that additional unaccounted factors may influence this outcome. Additionally, the f^2^ values provide insight into the effect size of each predictor on the dependent variables. Cohen (1988) suggests that f^2^ values of 0.02, 0.15, and 0.35 represent small, medium, and large effect sizes. The results indicate that the effect sizes vary across predictors, with some showing only marginal influence while others exhibit moderate explanatory power.

**TABLE 4:** Quality criteria of the model.

Variable	*R* ^2^	*R*^2^ adjusted	SD	T-statistics
Resilient coping	0.292	0.290	0.032	9.061***
Core self-evaluations	0.025	0.024	0.012	2.022*
Help-seeking attitudes	0.140	0.138	0.024	5.894***
Peer support	0.013	0.012	0.009	1.515*ns*
Suicidal behavior	0.042	0.039	0.013	3.159**

*N = 987. Significance levels:

***p < 0.001,

**p < 0.01, *p < 0.05*, ns, not significant.

In addition to these coefficients, overall model quality was assessed using key PLS-SEM fit indices. The standardized root mean square residual (SRMR = 0.054) and the normed fit index (NFI = 0.91) both met the conventional thresholds (SRMR < 0.08; NFI > 0.90), indicating that the proposed model provides an acceptable fit to the observed data. These indices, together with the R^2^ and f^2^ statistics, support the adequacy of the model and the robustness of its structural relationships.

As expected, the adjusted R^2^ values are slightly lower than the original *R*^2^ values due to the correction for model complexity. The significance tests indicate that the explained variance for Resilient Coping, Core Self-Evaluations, Help-Seeking Attitudes, and Suicidal Behavior is statistically significant. However, the variance explained in Peer Support is not statistically significant, suggesting that other factors might better account for its variance.

### Effect size (f^2^) matrix

Table 5 presents the f^2^ values, which quantify the relative contribution of each predictor to the variance explained in the dependent variables to assess the effect size of individual predictors.

**TABLE 5:** Effect size (f^2^) matrix.

Predictors	Outcomes				
	Coping strategies	Core Self-evaluations	Help-seeking behavior	Peer support	Suicidal behavior
Bully victimization	0.026	0.025	0.024	0.013	0.013
Core self-evaluations	0.114	–	0.036	–	0.019
Peer support	0.050	–	0.020	–	0.000

The f^2^ values further clarify the relationships among the variables, allowing for a more detailed interpretation of effect sizes. The small to moderate effect sizes suggest that while the predictors explain the variance of the dependent variables, additional factors may need to be considered to improve the model’s explanatory power.

## Discussion

The present study aimed to examine the psychological consequences of bullying victimization among Chinese adolescents, focusing on its associations with suicidal behavior, help-seeking attitudes, and resilient coping. By analyzing these relationships, we provide a deeper understanding of the factors contributing to risk and protective mechanisms in the adolescent bullying experience.

### Bullying victimization predicts help-seeking attitudes

Contrary to expectations, the results indicate that bullying victimization is negatively associated with help-seeking attitudes among Chinese adolescents. Rather than encouraging victims to seek assistance, experiences of bullying may deter them from reaching out for support. This finding suggests that feelings of shame, fear of retaliation, and low trust in institutional support contribute to the reluctance of victimized adolescents to engage with formal or informal resources.

The psychological consequences of bullying victimization may shape help-seeking behaviors in complex ways. While some research suggests that internalizing symptoms such as depression and anxiety can motivate individuals to seek help, the present findings highlight how psychological distress may also lead to social withdrawal and avoidance (Chen C. et al., 2025; Lin et al., 2024). Victims often experience loneliness and social anxiety that undermine their confidence to disclose distress (Li et al., 2023).

Cultural factors rooted in Confucian values may further explain this pattern. The emphasis on emotional restraint, harmony maintenance, and “face-saving” norms discourages open discussions of distress and dependence on external help. As a result, seeking assistance may be perceived as a loss of self-control or a disruption of group harmony. Similar barriers have been observed in other East and Southeast Asian contexts, where stigma and collectivistic norms shape adolescents’ help-seeking behaviors (Setia Lesmana and Chung, 2024; Setiyani Subardjo et al., 2024).

Cross-cultural evidence also reinforces the idea that social and institutional climates condition help-seeking after bullying. Studies from Germany, the United States, and religious contexts report that victims’ expectations about support outcomes (e.g., shame, empathy, or dismissal) critically influence their willingness to seek help (Deikus and Vveinhardt, 2024; Grüne and Willems, 2024; Harrison-Pavlik and Cashel, 2025; Kim and Craig, 2024). These findings are consistent with the present results, underscoring the global relevance of psychosocial and contextual barriers (Chen S. S. et al., 2025).

At the institutional level, school climates that promote open communication and emotional safety can help counteract these barriers. Strengthening teacher–student relationships and peer support networks may foster a sense of trust that enables victims to seek assistance (Lin et al., 2024; Zhang X. et al., 2024). Programs that combine stigma reduction, mental health literacy, and culturally responsive counseling practices appear essential to promoting help-seeking as a normative and acceptable behavior among Chinese adolescents.

### Bullying victimization predicts resilient coping

Contrary to the initial hypothesis, the findings suggest that bullying victimization is negatively associated with resilient coping among Chinese adolescents. Rather than fostering resilience, experiences of victimization appear to weaken the ability to engage in adaptive coping mechanisms. This indicates that prolonged exposure to bullying undermines the emotional resources needed for effective stress management.

While resilience is typically viewed as a protective factor, its function in the context of bullying victimization appears conditional and context-dependent. Some studies show that resilience can mitigate the adverse consequences of victimization, allowing adolescents to maintain psychological stability (İme and Çakır, 2024). However, resilience may not develop spontaneously in hostile environments; it requires external reinforcement through supportive social contexts and accessible coping models.

The finding that resilient coping exhibited the highest explained variance (R^2^) in the model highlights its central role as a potential target for intervention. This suggests that resilience-based programs could be especially effective in reducing the psychological harm of bullying. School-based initiatives that emphasize emotional regulation, cognitive reframing, and peer connectedness have been shown to strengthen resilience and prevent maladaptive coping (Yang et al., 2023; Wang et al., 2024).

Moreover, studies from Asian settings underline that resilience is closely linked to cultural values and social responsibility. In collectivistic contexts, adolescents often draw coping strength from group belonging and moral duty rather than from individual self-assertion (Setia Lesmana and Chung, 2024; Setiyani Subardjo et al., 2024). This cultural dimension implies that interventions should integrate both psychological and socioethical components, encouraging empathy, reciprocity, and community support.

Overall, these findings challenge the assumption that bullying victimization promotes resilient coping. Instead, they emphasize the importance of systematic efforts to cultivate resilience through supportive school environments and culturally grounded mental health education. Targeting resilience as a core intervention pathway may represent the most effective strategy for buffering adolescents against the long-term psychological consequences of bullying.

### Peer support and core self-evaluations as mediators

The mediation analysis highlights the crucial role of peer support and core self-evaluations in shaping the psychological impact of bullying victimization. Core self-evaluations significantly influence help-seeking attitudes and resilient coping, demonstrating that higher self-worth and emotional stability can buffer the adverse effects of victimization. Adolescents with more positive self-perceptions are better equipped to seek support and employ adaptive coping mechanisms, reinforcing the importance of self-efficacy, self-esteem, and emotional regulation skills. Similarly, peer support was positively associated with help-seeking attitudes and resilient coping, underscoring the protective role of strong social networks in mitigating the consequences of bullying.

While peer support proved to be an important factor in shaping adolescent coping mechanisms, it did not significantly mediate the relationship between bullying victimization and suicidal behavior. This absence of mediation may reflect the nature of peer relations in Chinese school settings, where friendships tend to be less emotionally expressive and more activity-based, limiting the degree to which peers provide intimate or crisis-related support. In collectivistic educational environments, adolescents often prioritize group harmony and academic achievement over emotional disclosure, which can reduce the protective impact of peer relationships on suicidality (Setia Lesmana and Chung, 2024). Other psychological mechanisms, such as depression, anxiety, and emotional dysregulation, may therefore play a stronger role in explaining the pathway between victimization and suicidality (Zhou et al., 2024; Santoso, 2024).

Cross-cultural findings similarly suggest that the effectiveness of peer support depends on cultural norms governing emotional expression and social reciprocity. In Western contexts, peer support often involves open emotional exchange, whereas in East Asian settings, mutual assistance is expressed through shared activity or silence, which may offer companionship but limited emotional buffering (Grüne and Willems, 2024; Kim and Craig, 2024). These cultural nuances may help explain the weaker mediating effect of peer support observed in the present study.

The mediation analysis further demonstrates that core self-evaluations play a pivotal role in fostering resilient coping among bullying victims. This finding highlights that self-perception is not merely a psychological construct but also a moral and developmental one—reflecting adolescents’ growing sense of agency, self-respect, and responsibility in confronting adversity. Resilience, when supported by strong self-efficacy and positive self-worth, has been shown to mediate the relationship between adverse childhood experiences and suicidal risk (Peng et al., 2024). This suggests that interventions aimed at strengthening self-esteem, emotional resilience, and ethical self-awareness could serve as protective measures against the negative psychological effects of bullying victimization.

The findings also highlight the variance explained in different psychological outcomes (Table 4). Resilient coping exhibited the highest explained variance, suggesting that the predictors significantly contribute to this outcome. Help-seeking attitudes also showed substantial explained variance, reinforcing the influence of core self-evaluations and peer support in shaping willingness to seek assistance. However, suicidal behavior displayed a lower R^2^ value, indicating that additional unaccounted factors, such as underlying psychiatric conditions or personal trauma, may drive this outcome. Notably, the variance in peer support was not statistically significant, suggesting that broader institutional and familial contexts—such as teacher responsiveness, parental involvement, and school policy—may play a more decisive role than peer dynamics alone.

Overall, these findings emphasize the importance of fostering supportive environments and strengthening self-perceptions to enhance adolescent coping mechanisms. While peer support and core self-evaluations contribute significantly to help-seeking and resilience, their limited buffering effect on suicidality underscores the need for comprehensive, multi-layered interventions that integrate psychological, educational, and cultural dimensions of adolescent wellbeing.

### Limitations of this study and suggestions for future research

While this study provides valuable insights into the relationships among bullying victimization, suicidal behavior, and help-seeking attitudes among Chinese adolescents, several limitations should be acknowledged. These limitations highlight areas for caution in interpreting the findings and suggest directions for future research.

First, the study employed a convenience sampling strategy, which may limit the generalizability of the findings beyond the sampled population. Participants were recruited from middle schools, and their experiences may not fully represent adolescents in other regions of China, particularly those in rural or socioeconomically disadvantaged communities (Mendez-Bustos et al., 2024). Future research should consider using random sampling to ensure broader representation and enhance external validity.

Second, data collection relied on self-reported measures, which may be subject to response biases, including social desirability and recall biases. Adolescents may have underreported or overreported their experiences with bullying, suicidal thoughts, or help-seeking behaviors due to stigma or personal discomfort (Barboteo et al., 2025). While the anonymous web-based survey likely reduced these biases, future studies should incorporate multi-informant assessments, such as reports from parents, teachers, or peer nominations, to improve the reliability of the data.

Third, the study’s cross-sectional design precludes any causal inferences regarding the relationships among bullying victimization, suicidal behavior, and help-seeking attitudes. Although significant associations were identified, the study cannot establish whether bullying victimization directly leads to suicidal ideation or whether other moderating or mediating factors contribute to this relationship. Future research should employ longitudinal designs to track changes in these variables over time and better understand the developmental trajectories of at-risk adolescents.

Additionally, while the study adhered to rigorous ethical safeguards to ensure participant wellbeing, the assessment of suicidal behavior remains a sensitive issue (Garcia-Fernández et al., 2021). Although students received information about psychological support services, screening for suicidal ideation through self-report measures alone may not be sufficient for identifying adolescents in immediate crisis. Future studies could integrate clinical interviews or real-time risk assessment tools to enhance at-risk students’ detection and intervention strategies.

Furthermore, the study primarily focused on individual-level factors such as coping mechanisms, self-evaluations, and peer support, without extensively examining broader socio-cultural and institutional factors that may influence bullying experiences and help-seeking behaviors. Future research should explore the role of school policies, family dynamics, and cultural attitudes toward mental health and victimization, as these may significantly shape adolescents’ willingness to seek help.

Lastly, although well-validated, the study’s measures were originally developed in Western contexts and later adapted for use in China. While previous studies have supported their reliability and validity in Chinese adolescent populations, future research should further examine cultural differences in the interpretation of these constructs, particularly regarding attitudes toward professional psychological help-seeking, which may be influenced by traditional beliefs and stigma surrounding mental health services.

In addition, the present research did not explicitly address the legal and ethical frameworks that govern school responsibility in protecting students from bullying and psychological harm. Future studies should integrate these dimensions by examining how educational policies, teachers’ duty of care, and ethical guidelines for mental health professionals interact with adolescents’ lived experiences of victimization. Such integration would allow for a more comprehensive understanding of the shared moral and institutional obligations to safeguard students’ wellbeing within both psychological and legal domains.

### Practical, ethical, and legal implications

The findings of this study offer important insights for educators, counselors, and policymakers seeking to reduce the psychological harm caused by bullying victimization among adolescents. Although some of the observed effect sizes were small (see Table 5), even modest associations can have substantial implications when scaled to school-wide contexts. A small f^2^, for example, may indicate that single variables explain a limited proportion of variance, yet they represent actionable entry points for intervention—especially in prevention programs that target large populations over time. This supports the implementation of early, multi-component strategies addressing both individual resilience and systemic support structures.

From a practical standpoint, the results highlight the central role of resilience and self-evaluation in adolescent adjustment. School-based programs that incorporate emotional regulation training, peer cooperation, and cognitive reframing can strengthen students’ capacity to cope adaptively with bullying. Teachers and counselors should receive specialized training to recognize signs of psychological distress and to guide victims toward professional help. Peer-based initiatives, such as student mentoring or peer advocacy systems, should not only provide emotional comfort but also act as bridges to adult and institutional sources of support, thereby extending the scope of peer involvement beyond informal reassurance.

Ethically, the findings underscore the duty of care that educators and mental health professionals hold toward adolescents in distress. Creating emotionally safe environments requires not only empathy but also adherence to ethical principles of confidentiality, non-maleficence, and respect for autonomy. Training modules in moral education and professional ethics should integrate case-based discussions on bullying, emotional disclosure, and student protection, helping educators to navigate the balance between safeguarding and respecting students’ privacy.

Legally, this study points to the importance of aligning psychological interventions with existing child protection frameworks and educational policies in China. National and regional regulations, including the “Law on the Protection of Minors” and the “Guidelines for the Prevention and Handling of School Bullying,” establish explicit obligations for schools to prevent, identify, and respond to bullying incidents. The present findings reinforce these legal imperatives by demonstrating how supportive school climates and accessible counseling systems can mitigate the psychological consequences of victimization. Integrating empirical evidence from psychology into policy enforcement can therefore strengthen accountability and improve implementation fidelity across educational institutions.

Finally, culturally sensitive interventions aimed at reducing stigma, increasing mental health awareness, and strengthening formal and informal support systems are essential for fostering more positive help-seeking behaviors among adolescents. Building on these principles, collaboration among schools, families, and community agencies should be prioritized to ensure an integrated response to bullying that respects both the legal rights and emotional needs of young people.

## Conclusion

This study underscores the significant psychological consequences of bullying victimization among Chinese adolescents, particularly its association with suicidal behavior, help-seeking attitudes, and resilient coping. The findings indicate that victimization increases the risk of suicidality, discourages help-seeking behaviors, and diminishes resilient coping mechanisms. Importantly, peer support and core self-evaluations serve as critical mediators that can either mitigate or exacerbate these effects. While strong self-evaluations promote adaptive coping and help-seeking, peer support does not significantly mediate the relationship between victimization and suicidal behavior, suggesting that additional factors contribute to this outcome.

Beyond individual mechanisms, the study contributes to a broader understanding of how cultural values, ethical responsibilities, and institutional frameworks shape adolescents’ responses to bullying. The influence of Confucian norms of restraint and harmony, together with the legal and moral obligations of schools to protect students’ wellbeing, underscores that psychological resilience must be supported within safe and ethically responsive educational systems.

The study highlights the urgent need for targeted interventions at multiple levels. Schools should integrate structured programs to enhance emotional regulation and social support, while counselors and mental health professionals must provide specialized services for victims. Parents play a vital role in fostering communication and emotional resilience, while societal efforts should focus on policy-based solutions to address bullying prevention and mental health support. Collaborative, culturally sensitive interventions that align psychological, ethical, and legal perspectives are essential to ensure a holistic response to bullying victimization. Future research should investigate additional mediating mechanisms and intervention strategies to enhance outcomes for adolescents who have been victimized. By implementing evidence-based and ethically grounded interventions, educators, counselors, parents, and policymakers can create safer, more inclusive school environments that foster adolescent wellbeing and social responsibility. The integration of psychological, ethical, and legal perspectives achieved in this study advances a more comprehensive view of adolescent wellbeing and accountability, with implications for both practice and educational policy.

## Data Availability

The raw data supporting the conclusions of this article will be made available by the authors, without undue reservation.
